# Circularly Polarized Hybrid Dielectric Resonator Antennas: A Brief Review and Perspective Analysis

**DOI:** 10.3390/s21124100

**Published:** 2021-06-15

**Authors:** Rajasekhar Nalanagula, Naresh K. Darimireddy, Runa Kumari, Chan-Wang Park, R. Ramana Reddy

**Affiliations:** 1 Department of Electrical and Electronics Engineering, BITS-Pilani, Hyderabad 500078, India; rajasekhar434@gmail.com (R.N.); runakumari@hyderabad.bits-pilani.ac.in (R.K.); 2 Department of Mathematics, Computer Science and Engineering, University of Quebec, Rimouski, QC G5L 3A1, Canada; chanwang_park@uqar.ca; 3 Department of Electronics and Communication Engineering, JNTUA College of Engineering Pulivendula, Andhra Pradesh 516390, India; profrrreddy@yahoo.co.in

**Keywords:** CP radiation, microstrip antenna, dielectric-resonator antenna, hybrid dielectric resonator antenna, wide-band antennas, multi-functional antennas

## Abstract

Recently, it has been a feasible approach to build an antenna, in view of the potential advantages they offer. One of the recent trends in dielectric resonator antenna research is the use of compound and hybrid structures. Several considerable investigations have been already underway showing quite interesting and significant features in bandwidth, gain, and generation of circular polarization. A critical review on a journey of circularly polarized hybrid dielectric resonator antennas is presented in this article. A general discussion of circular polarization and DR antennas are provided at the forefront. Evolution, significant challenges, and future aspects with new ideas in designing hybrid dielectric resonator antennas are indicated at the end of the review. State-of-the-art advances and associated design challenges of circularly polarized hybrid DR antennas and related empirical formulas used to find resonance frequency of different hybrid modes produced are discussed in this paper.

## 1. Introduction

The radiation mechanism of various types of antennas and their radiation fundamentals, focusing on the concepts of circular polarization, are comprehensively discussed in [1,2]. Circularly polarized (CP) antennas can offer a consistent or reliable system connection between the transmitter and receiver, since the polarization of the antennas is always associated. In and around the intended resonant frequency, two modes orthogonal to each other with a phase-shift of 90° are desired for circular polarization. Sometimes, the horizontal and vertical field components of a communications link are highly uncorrelated. Therefore, using receiver antennas with the same phase center and orthogonal polarizations can avoid location-induced phase variation.

Generally, a circularly polarized antenna should have the following essential characteristics,

- Nearly equal magnitudes for two orthogonal modes or polarizations and equal radiation pattern shapes.
- Nearly ±90° difference in phase over a wide-bandwidth and wide-beam width.
- Small axial ratio (close to 0 dB i.e., <3 dB or numerical 1) over a wide-AR bandwidth and wide-beam width.

The polarization of a wave is expressed in terms of the figure traced as a function of time by the extremity of the E-field vector at a fixed location in space, the sense in which it is traced, and observed along the direction of propagation. The instantaneous electric field of a uniform plane wave pointed in the negative z-axis is expressed by Equation (1):

(1) $$E_{i}\left(z,\;t\right)=E_{ix}\left(z,t\right)a_{x}+E_{iy}\left(z,t\right)a_{y}$$

The instantaneous E-field components of *x* and *y* are correlated to their complex quantities by the following Equations (2) and (3):

(2) $$E_{ix}\left(z,\;t\right)=E_{ix}\;Cos\left(wt+\beta z+\Phi_{ix}\right)a_{x}$$

and

(3) $$E_{iy}\left(z,\;t\right)=E_{iy}\;Cos\left(wt+\beta z+\Phi_{iy}\right)a_{y}$$

The ***E_ix_*** and ***E_iy_*** are amplitudes, and *Φ_ix_* and *Φ_iy_* are the corresponding phases in *x* and *y* directions respectively, *w* is the angular frequency and *β* is the propagation constant.

- For linearly polarized (LP) plane wave, the difference of phase angle between the *x* and *y* components must be:
  (4) $$\delta \Phi =\Phi_{iy}-\Phi_{ix}=m\pi ,\;where\;m=0,1,2,\ldots \ldots \;$$
- For the circularly polarized wave, the magnitudes of the *x* and *y* components are equal (i.e., $E_{ix}=E_{iy}$), and difference in phase angle is odd multiples of 90°, and it is mathematically expressed as:
  (5) $$\delta \Phi =\Phi_{iy}-\Phi_{ix}=\langle \begin{array}{c} +\left(2m\pi +\frac{\pi }{2}\right)\;for\;Right\;Hand\;CP\\ or\\ -\left(2m\pi +\frac{\pi }{2}\right)\;for\;Left\;Hand\;CP \end{array}$$
- If δΦ does not satisfy Equation (1) or $E_{ix}\neq E_{iy}$ then the wave is elliptically polarized.

In this review article, conditions for CP radiation, different methods of generating circular polarization in dielectric resonator antennas are studied and analyzed by considering a few standard articles in the open literature. Historical study of microstrip and dielectric resonator-based antennas; the important milestones and significant features of dielectric resonator antennas are discussed and reviewed respectively in Section 2. Various aspects and techniques involved in generating circularly polarized radiation for DR-based antennas are studied, in order to recognize the associated challenges. Consequently, the evolution of dielectric resonator-based hybrid antennas with CP radiation techniques is reviewed comprehensively in Section 3. This article’s intention and primary focus are on various design perspectives and the progress of circularly polarized hybrid dielectric resonator antennas.

## 2. Historical Review of DR Antennas and CP Methods

Even though Deschamps was the first to introduce the concept of low-profile microstrip radiators in 1953 [3], it was after 20 years that the original patch antenna was practically developed by Munson [4,5] i.e., in the year 1974. The numerous benefits of microstrip antenna, such as less weight, low volume, easy to integrate with the printed circuit board (PCB) technology, are explored to design different patch configurations and feed mechanisms for numerous applications [6,7,8,9,10]. In today’s modern world, the essential need and demand for low-profile and compact antennas to integrate with mobile and personal communication devices has drawn the patch antennas to the forefront.

The radiation mechanism of DRAs is quite different from microstrip radiators with similar excitation methods. The electromagnetic energy fed to the DR is confined to the dielectric material, and the radiating mode of that material is excited, to act as a resonator. In another way, the confined EM energy is controlled through the design of DR, and the energy leaks from the resonator. A wide range of dielectric constants, starting from 8 to over 100 are used. The potential of DRAs was its high-frequency operation and wide impedance bandwidth with sufficient gain, compared to the metallic patch antennas. Several modes are excited in DRA, similar to the short magnetic or electric dipole antenna radiation patterns. The cause for radiation in the microstrip antenna is the two narrow radiating edges, while the DRA radiates through the entire DR external surface, excluding the grounded area. The reduction of surface-waves is an important advantage of the DRA in conjunction with a patch antenna. However, excitation schemes of the DRA and patch antennas are quite similar, and the behavior of both is similar to that of resonant cavities. The dielectric wavelength is lesser than the free-space wavelength by an element of 1/√(ε_r_), so increasing ε_r_ both of them can be made smaller.

Additionally, all the feeding methods used for the patch antenna can also be applied for the DRA. The dimensions of a DRA are of the order λ_0_⁄(√(ε_r_) or less, where λ_0_ is the free space wavelength, and ε_r_ is the material dielectric-constant. The radiation Q-factor and frequency of resonance can be altered by the aspect ratio of the DRA for a fixed value of ε_r_. With the absence of surface waves, the conductor losses are minimum in DRA and high radiation efficiency can be achieved [11]. Generally, DR is mostly used in traditional applications such as microwave circuits, oscillators and filters [12]. The DR was typically considered as an energy storage element instead of an antenna or radiator [13,14]. Even though Richtmyer first proposed the radiation concept of Dielectric Resonators in 1939, the systematic study and experimentation were carried out by Professor S. A. Long in 1983. Many communication systems’ frequency range of interest had increasingly advanced to the near-millimeter and millimeter frequency range (100–300 GHz). The conductor loss at this frequency range for microstrip antennas becomes high, and it yields a reduction in the efficiency of the radiators. After the cylindrical DRA, Long and his colleagues subsequently investigated the rectangular [15], cylindrical [16], and hemispherical [17] DRAs. Other shapes are also investigated, comprising the triangular [18], spherical-cap [19], cylindrical-ring [20], and mushroom-shaped [21] DRAs. Consequently, few review papers are reported in the open literature focusing on various aspects, such as general design equations of DRAs for bandwidth, frequency, and equivalent mode theory [22], study of broadband DRAs [23], design advances in dielectric resonator-based ultra-wideband monopole antennas [24], a historic study of DRAs and its state-of-the-art based on the radiation parameters [25,26,27], circularly polarized DRAs [28], modeling DRAs using numerical methods [29], application-oriented DRAs [30], various CP methods in DRAs [31], improvement of impedance bandwidth in DRAs [32], and design advances in various types of CP antennas [33].

A few glimpses of circularly polarized DR antennas are studied to acknowledge the design aspects responsible for CP radiation. A pair of orthogonal HE_11δ_ modes of a cylindrical ring DRA in phase-quadrature is presented for CP radiation. Mongia et al. reported a DRA with a 3 dB AR (Axial Ratio) beamwidth of 1000 and a minimum AR value of 0.5 dB [34]. A single point probe-fed elliptic DRA is presented for CP radiation with wideband properties and it provides 3.5% of CP bandwidth and 14% of RL bandwidth [35]. A parasitic strip is placed on the adjacent wall of the dielectric resonator to stimulate degenerated modes using finite difference time domain (FDTD) analysis to address the linear and circular polarized modes [36]. A new comb-shaped CP-DRA [37] is proposed, and it offers about 4% AR bandwidth with a gain of 3.5 dBi. A quadruple strip-fed cylindrical DRA using two orthogonal hybrid couplers reported for wideband circular polarization applications [38]. The circularly polarized C-shaped DRA [39] has 19% of AR bandwidth and is enhanced to 50% using a short-circuit microstrip. A compact wideband rectangular DRA based on perforations and edge grounding is investigated in [40]. To reduce the Q-factor (i.e., inversely proportional to bandwidth), square slots (perforations) are bored equivalently all across the DRA. The overall occupancy of DRA is reduced by cutting slots and an edge grounding technique in rectangular DRA. A wideband dual segmented DRA is proposed for X-band applications. An S-shaped slot is used to couple the DR elements, and it offers 37.5% (7.66 to 11.2 GHz) fractional bandwidth with a peak gain of 6 dBi [41]. A wideband CP pixelated DRA is analyzed using a real-coded genetic algorithm (GA), and it is coupled through a narrow slot on the ground plane [42]. A combination of an L-shaped microstrip line and a conformal strip is used as feed to excite two orthogonal modes in two cubical DR elements for CP radiation and wideband applications [43]. A combination of two L-shaped slots is used for two configurations to obtain dual-sense polarized triband and quadband DR antennas [44] for multi-functional applications. Two wideband cylindrical DR antennas loaded on two configurations of phase delay lines (PDL) are presented [45] for CP radiation at 2.4 GHz applications. The CP radiation with two orthogonal modes is possible due to stub-loaded 90° and 180° PDLs. The wide impedance bandwidth and low spurious feed radiation with minimized surface wave losses are advantages of DRA compared to patch antennas. The requirement of DRAs with wide AR bandwidth and wide impedance bandwidth is increasing day-to-day.

## 3. Classification and Progress of Dielectric Antennas

**Figure 1:**
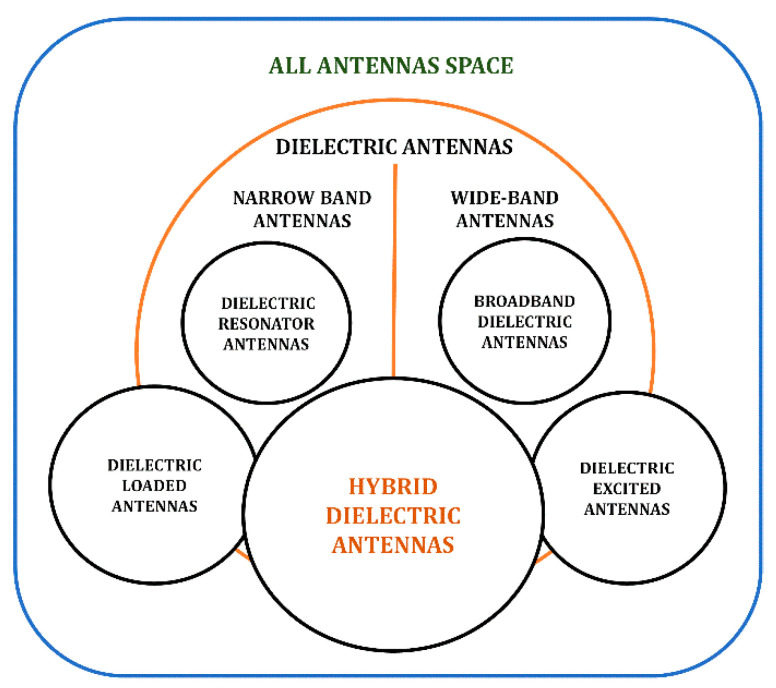
Venn Diagram representation for dielectric antenna technology.

In defining the radiation properties of various types of dielectric antennas, shape and relative permittivity of dielectric material are the significant parameters [46,47,48,49,50], which add up a degree of freedom compared to microstrip radiators. A Venn diagram based on technological advancements in the field of dielectric antennas is presented in Figure 1.

The past review studies of the dielectric resonator antennas have been summarized in Table 1 to highlight the various significant radiation and design aspects. The stored energy inside the dielectric is exceptionally high and it is difficult for external objects to detune the device [51,52,53]. DR can radiate from all surfaces, rendering high radiation efficiency and low Q-factor. Since its birth in the early 1980s, there has been a steady research progress in this area over the years. The bandwidth of a resonant device is a function of its loaded Q, which is controlled by the way of energy, is coupled in and out of the resonant device is under the designer’s control. The unloaded Q is a measure of the internal losses in the device. A device with a high unloaded Q may be used to create an antenna structure with low loaded Q and wide impedance bandwidth. Broadband dielectric antennas can be obtained by suitably configuring the feed structure [54,55,56].

The primary radiating component of the *Dielectric Loaded Antennas* is a conducting element and the dielectric modifies the medium, imparting significant performance advantages [57,58,59,60,61]. Dielectric antennas can be used to excite parasitic copper antennas or vice versa. In this class of antennas, often the conductor forms the major radiating part of the antenna. There are bandwidth advantages in this dielectric–copper hybrid approach of the *Dielectric Excited Antennas* [62,63,64,65]. Dielectric resonator-based loaded antennas and excited antennas come under the category of *Hybrid Dielectric Resonator Antennas*. Mongia et al. introduced a combination of the grounded metallic post at the center of cylindrical DR elements to produce the lowest order mode for which the overall size of the DRA has been significantly reduced [66].

**Figure 2:**
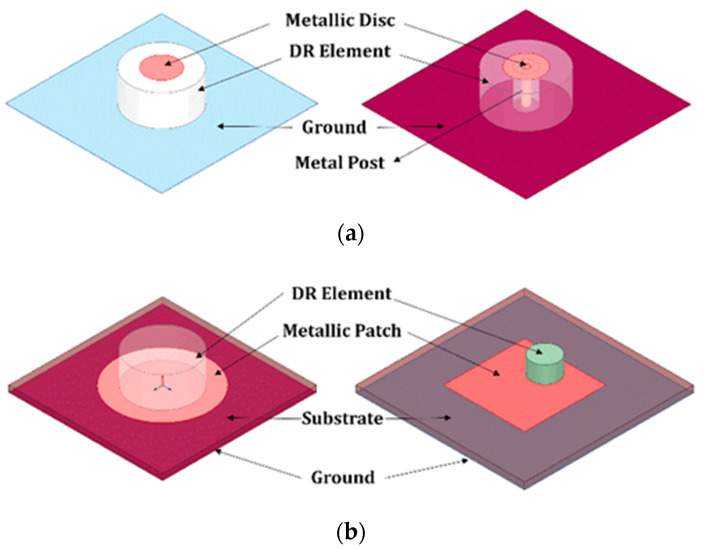
A few examples of hybrid dielectric resonator antenna technology. (**a**) microstrip disc loaded dielectric antennas, and (**b**) dielectric loaded microstrip patch antennas.

Similarly, the frequency shift is also possible with the circular metallic disk on the top of the DR element, either isolated or grounded using metallic posts reported by Li et al. [67]. A combination of monopole and ring-shaped DRA forming hybrid structures [68] is developed for ultra-wideband applications with omnidirectional patterns. Further, a microstrip loaded with small cylindrical DRA combination with a wide impedance bandwidth of 10% is reported [69], compared to microstrip alone. Ittipiboon et al. reported a linearly polarized dielectric-loaded antenna with a suspended microstrip in the air to achieve wide impedance bandwidth [70,71]. Various configurations of hybrid dielectric antennas have been illustrated in Figure 2. Several designs of linearly polarized wideband [72,73,74,75,76,77,78,79,80,81,82,83,84,85,86,87,88,89,90] and multi-functional [91,92,93,94,95,96,97,98,99,100,101,102,103,104,105] hybrid dielectric resonator antennas are reported in the open literature. Dielectric resonator-based hybrid antenna structures are the recent research trend and focused area, as they can have the combined advantages of microstrip and dielectric resonator (DR) antennas. In the following discussion, existing DR-based hybrid structures explicitly with CP-radiation techniques are reviewed comprehensively.

## 4. Circularly Polarized DR Based Hybrid Antennas

### 4.1. Wideband Hybrid Antennas with CP Radiation

Several CP radiation techniques and respective design methods have been implemented independently for microstrip and dielectric resonator antennas. However, CP radiation techniques for hybrid dielectric resonator antennas over a wide-bandwidth are challenging [106]. A compact dielectric-loaded aperture-coupled microstrip antenna is reported for L-band mobile satellite applications with CP radiation. Two dielectric-inserts [107] along the edges of the square patch are positioned for the required reduced frequency shift of 30% to the lower-side, and the cross-slot aperture creates the two-orthogonal modes desired for CP radiation with 2.5% of 3 dB AR bandwidth compared to traditional CP square patch. Similarly, four dielectric inserts [108] are used under the square patch positioned at the edges with a cross-slot feed to obtain the CP radiation. A considerable reduction of antenna size is possible by inserting dielectric blocks beneath the patch, while the desired bandwidth and the axial ratio are also achieved for L-band applications [109]. A strip-line fed compact rectangular DR-based antenna with a top-loaded rectangular patch of various aspect ratios is reported for circularly polarized radiation [110]. A suitable selection of aspect ratio for the top-loaded rectangular patch, a fundamental resonant mode of TE_111_ of the rectangular DR antenna, can be divided into two orthogonal degenerated modes (TE^x^_111 and_ TE^y^_111_), which leads to circular polarization. A novel Hybrid DRA consisting of four rectangular slots on the ground-plane acts as an aperture for the DR element [111]. The wide bandwidth of 500 MHz (1130–1630 MHz) with CP radiation is achieved at boresight with a wide AR beam-width of 100 degrees. A compact Hybrid DRA composed of cylindrical DR elements and four arc-shaped slots arranged sequentially on the ground plane, which acts as an aperture to the DR is proposed [112] for GPS and GNSS applications. In this case, the CP radiation is responsible due to the geometrical arrangement of slots and the feed network with four strip-lines. A high-gain single element hybrid DRA consisting of the microstrip and the elliptic-shaped dielectric ring is proposed [113] for millimeter-wave frequency applications. The combination of microstrip and elliptical dielectric ring is responsible for high gain and an inverted T-shaped slot is responsible for CP. The fine-tuning of the AR bandwidth and gain is possible, and can be achieved by ring-shaped DR. The maximum impedance bandwidth of 12% and an AR bandwidth of 10% with a measured gain of over 9dBi is obtained over the entire frequency band.

**Figure 3:**
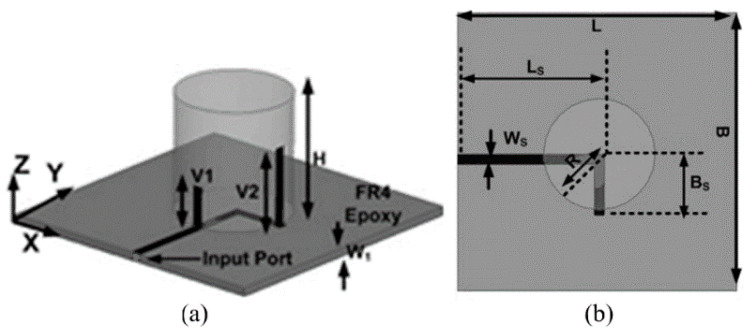
(**a**) 3D-view and (**b**) Top-view of hybrid structure (Reference [116]).

**Figure 4:**
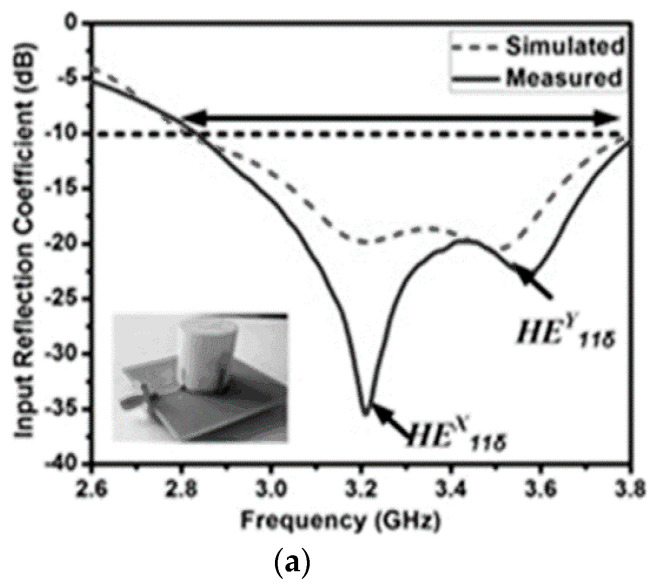

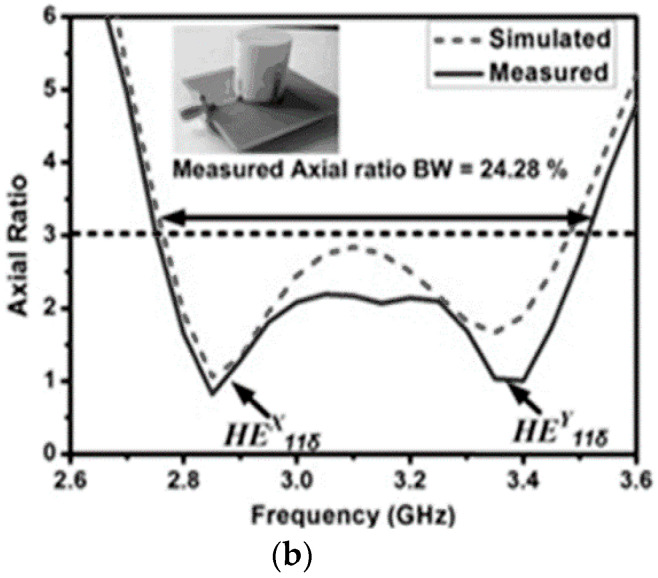
Representation of simulated and measured (**a**) S11 (**b**) AR plots, along with orthogonal modes. (Reference [116]).

**Figure 5:**
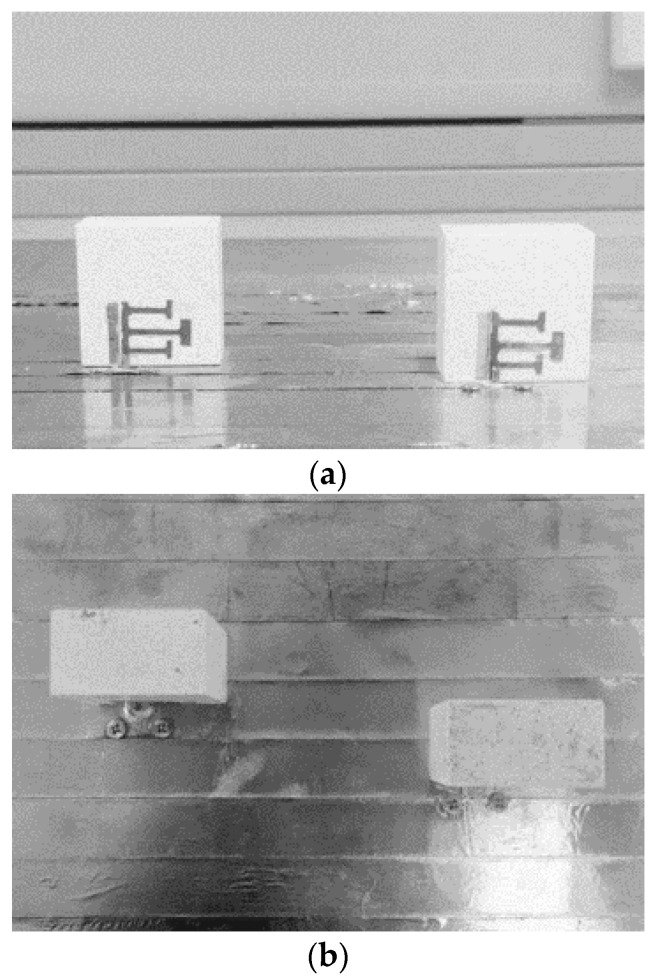
Diagonally arranged two-element multiple inputs multiple output (MIMO) hybrid structure (**a**) Side view, (**b**) Top view (Reference [117]).

A modified cross-slot is concurrently acting as a radiator as well as the feeding element to the DR antenna. The resonances of modified cross-slot and DR elements are combined to form a wideband CP radiated hybrid DRA [114] with a 3 dB AR bandwidth of 24.6% (2.25–2.88 GHz) and 10 dB return-loss bandwidth of 28.6% (2.19–2.92 GHz). A hybrid structure is formed, combining a stair-shaped DR element with an open-ended slot [115] on the ground plane, which is reported for wideband CP radiation. The open-ended slot on the ground plane is responsible for a lower frequency of resonance (at 4.5 GHz) and, in turn, a wide axial-ratio bandwidth is obtained. By varying different parameters of the hybrid structure, a wide impedance bandwidth of 71.7% (3.844–8.146 GHz) and an AR band-width of 46% (4.15–6.63 GHz) is obtained. A simple feed network along with two vertical strip-lines are arranged [116] as shown in Figure 3 to produce orthogonal fields of hybrid HE^x^_11$_ and HE^y^_11$_ modes (presented in Figure 4) in the cylindrical DR element for wide CP radiation with AR bandwidth of 24.6% (2750–3520 MHz). The length of the vertical strip-lines along the cylindrical surface of the DRA controls the LHCP and RHCP radiation. A circularly polarized multiple inputs multiple output (MIMO) hybrid antenna [117] is formed by a parasitic patch, and the conformal strips along the sidewalls of two identical rectangular DR elements are shown in Figure 5. The single element of the proposed structure produces linear polarization at the desired frequency, and if both the elements are arranged diagonally, CP fields are created with two orthogonal modes. The AR bandwidth offered by the MIMO structure is 20.82% in the frequency band of 3.58–4.40 GHz. An electromagnetically coupled hybrid antenna comprises a hexagonal split-ring slotted hexagonal patch loaded, with a parasitic rectangular dielectric block at the radiating edge of the patch for wide-band CP radiation applications [118]. The performance analysis and features of wide-band DR-based hybrid antennas are summarized in Table 2. By considering the relative permittivity (ε_r_) of the dielectric resonator in the range 9 to 80, the effective height (H_eff_) of hybrid DR antenna can be in the range 0.0118λ < h < 0.034λ, while sustaining 10 dB RL and 3 dB AR bandwidths to the higher values. The ground plane dimensions determine the overall volume hybrid DR antenna. The size of the ground plane was chosen appropriately and not to reduce the whole volume of the antenna. The dimensions of the ground plane are kept as low as 0.37λ in the reported literature, and it can be minimized, if the antenna designer is ready to compromise bandwidth and gain performance.

### 4.2. Multi-Functional Hybrid Antennas with CP Radiation

A combination of ring-shaped microstrip and cylindrical DR elements has been designed for dual functional applications. In this particular configuration, an independently operated microstrip-based annular ring and cylindrical dielectric resonators are used to form a hybrid structure [119], to produce dual CP-radiated bands at 4.2 GHz and 6.4 GHz, respectively, with the return-loss bandwidth higher than 6% at each band. By short-circuiting the annular ring to the ground, the perturbation of radiation patterns can be avoided after assembling both the resonators significantly. However, the shift in the resonant frequency due to the short circuit can be adjusted by modifying the annular ring dimensions. A dual CP hybrid DR antenna is formed [120] by introducing a zonal slot cut on a conducting-cavity, along with a DR element. The lower and upper bands are achieved by zonal slot and DR elements positioned on the side-wall and top of the cavity wall. An L-shaped probe is used to feed the zonal slot antenna in the cavity and an indirect cross-coupling slot is incorporated to feed the DR element. Two even cuts are introduced to achieve CP radiation fields, and another cut is introduced in the zonal slot to obtain the desired AR. Regular unequal lengths of cross-slots under the DR element introduces another perpendicular degenerated mode for CP fields. A cross-slot is used as an aperture feed and as a radiator to produce dual-CP-bands [121]. A combination of dual-C-shaped microstrips and two DR elements are arranged in a Z-shape, forming a hybrid structure [122] to achieve multi-functional bands with CP radiation. Five bands, including one CP band, are produced in the 1 to 9 GHz frequency range. The TE_01δ_ mode is produced due to a dual C-shaped patch and an equipoised top and bottom cylindrical DR elements responsible for orthogonal fields result in CP radiation. The performance characteristics of multi-functional DR-based hybrid antennas are presented comprehensively in Table 3. The feed, a combination of radiating elements in the hybrid structure and placement and orientation of the DR element is responsible for producing multi-functional resonant bands. As reported in Table 3, the gain in all functional bands depends on the appropriate dimension of the ground plane and the effective height of the hybrid DR antenna.

### 4.3. Dual-Sense Polarized Hybrid Antennas

**Figure 6:**
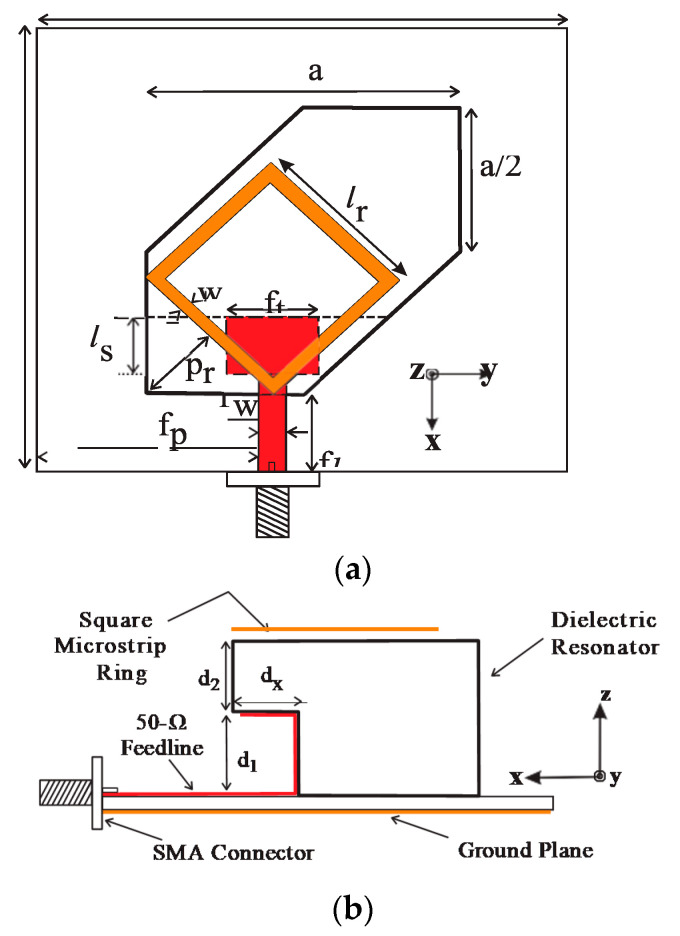
Configuration of dual-sense polarized hybrid DRA. (**a**) Top view, and (**b**) side view (Reference [128]).

Earlier, a combination of monopole and ring DR antennas are ensembled to produce wide bandwidth with Omni-directional patterns [123,124,125]. Similar designs can also have multiple bands with orthogonal polarizations with coax-feed and dual-feed techniques, respectively. Later, a few more hybrid antennas with a combination of microstrip and DR elements are proposed for multi-functional dual-sense polarizations, which include: a circular-ring patch with a reversed L-strip [126]; an irregular square-ring patch loaded DR element [127]; a tapered microstrip line feed; modified unequal sides of hexagonal DR loaded with the square ring [128] (shown in Figure 6); an L-shaped line stub feed incorporated in an irregular rectangular slot [129]; and a line feed hybrid DR antenna [130] with top-loaded inter-digital structure. The performance characteristics of dual-sense polarized DR-based hybrid antennas are summarized in Table 4. The combination of feeding mechanism and patch design beneath or on top of the DR element, and the shape of the DR element are responsible for multi-sense polarized radiations.

## 5. Formulation to Find Resonance of Various Modes Associated with Hybrid DRAs

In this section, a summary of empirical formulas used in the literature to find the fundamental mode of resonant frequency and various hybrid modes for hybrid rectangular and cylindrical DRAs are given in Equations (6)–(16).

For hybrid rectangular DRA [131], the analytical solution of fundamental resonance is given in Equations (6)–(8).

(6) $$f_{res}=\frac{C}{2\pi \sqrt{\in_{r,\;\;RDR}}}\;\sqrt{k_{x\;}{}^{2}+k_{y\;}{}^{2}+k_{z\;}{}^{2}}$$

(7) $$\frac{k_{x}\;a}{2}=\tan^{-1}\sqrt{\;\left(1-\frac{1}{\in_{r,\;\;RDR}}\right){\left(\frac{k_{0}}{k_{x\;}}\right)}^{2}-1}\;$$

(8) $$k_{0}=\frac{2\;\pi }{\lambda_{0}}=\frac{2\pi f_{res}}{C},\;\;\;\;\;\;\;k_{y}=\frac{\;m\pi }{b}\;,\;k_{z}=\frac{n\pi }{2d}$$

The “*a*”, “*b*”, and “*d*” are the dimensions of the rectangular DRA designated as length, width, and height, respectively. Similarly, to find the resonant hybrid (TE_01δ_ and HE_11δ_) modes in hybrid cylindrical DRA, the following Equations (9) and (10), respectively, are used along with the effective permittivity and height Equations (11) and (12).

(9) $$f_{r,{\;\mathrm{TE}}_{01\delta }}=\frac{2.327c}{2\pi d\sqrt{\in_{r,eff}+1}}\left[1.0+0.2123\frac{d}{H_{eff}}-0.00898{\left(\frac{d}{H_{eff}}\right)}^{2}\right]\;$$

(10) $$f_{r,{\;\mathrm{HE}}_{11\delta }}=\frac{6.321c}{2\pi d\sqrt{\in_{r,eff}+2}}\left[0.27+0.36\frac{d}{2H_{eff}}+0.02{\left(\frac{d}{2H_{eff}}\right)}^{2}\right]$$

If a multi-segmented antenna is considered, the resonance frequency will be affected by the layers of the substrate (*H_S_*) and dielectric (*H_D_*) materials. Accordingly, the effective height (*H_eff_*), and permittivity $\left(\in_{r,eff}\right)$ [132,133] of the hybrid CDRA are calculated by Equations (11) and (12):

(11) $$H_{eff}=H_{DR}+H_{Sub}$$

Similarly, the effective relative permittivity $\in_{r,eff}\;$in Equation (8) is given by:

(12) $$\in_{r,eff}=\;\;\frac{H_{eff}}{\frac{H_{DR}}{\in_{r,CDRA}}+\frac{H_{Sub}}{\in_{r,sub}}}$$

where “*d*” (D/2) is the radius of the cylindrical DR element.

The mathematical prediction of other hybrid radiating modes has been discussed qualitatively in [134], and they are calculated by Equations (13)–(16). The higher-order modes of HEM_11δ_ mode are HEM_11δ+1_ and HEM_13δ_. In the case of a cylindrical structure, the predicted resonant frequencies for these higher-order modes are given by the first-order Bessel function [135] and given by Equations (13) and (14). Guha et al. proposed [136] a new hybrid mode of HEM_12δ_ (Equation (15)) and predicted the resonance using HEM_11δ_ mode and aspect ratio of CDRA. The higher order mode of HEM_12δ_ is HEM_14δ_, and it is given in Equation (16).

(13) $$f_{r,{\;\mathrm{HEM}}_{11\delta +1}}=1.25\times f_{r,{\;\mathrm{HEM}}_{11\delta }}$$

(14) $$f_{r,{\;\mathrm{HEM}}_{13\delta }}=1.5\times f_{r,{\;\mathrm{HEM}}_{11\delta +1}}+\left(f_{r,{\;\mathrm{HEM}}_{11\delta +1}}-f_{r,{\;\mathrm{HEM}}_{11\delta }}\right)$$

(15) $$f_{r,{\;\mathrm{HEM}}_{12\delta }}=1.8\times f_{r,{\;\mathrm{HEM}}_{11\delta }}$$

(16) $$f_{r,{\;\mathrm{HEM}}_{14\delta }}=1.25\times f_{r,{\;\mathrm{HEM}}_{12\delta }}\;$$

## 6. Future Scope and Challenges

Based on the review, several techniques that can be implemented in the future are listed below to achieve wideband, multi-band, high gain, and circularly polarized hybrid DR-based antennas.

- Employing metamaterial concepts and magnetic LC resonators on the metallic patch, considering dielectric-loaded patch and metallic patch loaded on top of dielectric antennas.
- Various available fractal concepts can be employed on the patch as well as the DR elements particularly to achieve multifunctional bands of resonance.
- Existing bandwidth and gain enhancement techniques can be applied for the combined form of hybrid DR-based antennas.
- A combination of a metallic waveguide, microstrip, and DR antennas with a proper feeding mechanism can be developed for future radar applications.

Implementing multi-functional CP bands is a challenge for hybrid dielectric resonator antennas with desired gain over all the resonances.

## 7. Conclusions

A brief study and evolution of circularly polarized DR-based hybrid antennas and design challenges are discussed in this article. Various methodologies and insights into obtaining wide bands and multi-functional bands with CP radiation using DR-based hybrid antennas are addressed and reviewed. A perspective analysis and study of DR antennas with various CP radiation methods are presented. The empirical formulas that are being used and correlated in the open literature to identify different resonant modes, including the fundamental mode of resonance, are addressed in the process of designing various hybrid DR antennas. Table 1 presents the past review highlights and state-of-the-art in DR antennas. Table 2, Table 3 and Table 4 present the various methods of CP radiation in designing wideband, multi-band, and dual sense polarized DR-based hybrid antennas. Based on this review, a new line of work is highlighted with important future challenges and possibilities. The qualitative and quantitative information given in this review article is useful for engineers who are working on circularly polarized wideband and multiband hybrid DRAs.

## Figures and Tables

**Table 1:** Past review highlights of dielectric antennas.

Year	Past Review Highlights	Reference
1994	A detailed review of modes and various radiation parameters of DRAs of different shapes are discussed. A precise closed-form equation is presented to obtain fundamental resonant frequencies and bandwidth of a cylindrical DR.	[22]
2005	A brief review of broadband DRAs and design techniques for obtaining multi-resonant frequencies and how these can be combined to form broadband are discussed.	[23]
2006	Advancements and challenges in designing composite and hybrid dielectric resonator antennas are addressed.	[41]
2010	A detailed review of various types of monopole-based DR antennas and design methodologies are addressed for ultra-wideband applications.	[24]
2010	A comprehensive study of history and state-of-the-art design techniques in dielectric resonator antennas over the last 30 years are discussed.	[25]
2012	A study of recent developments of DR antennas for different radiation parameters and the use of decorative glass blocks as DR elements are presented.	[26]
2014	Latest developments in the design of dielectric resonator antennas in wideband, multi-band, and ultra-wideband categories have been addressed comprehensively.	[27]
2015	Various design and developments in methods of CP DRAs are addressed	[28]
2016	A review of numerical methods used to model the DR elements considering all the effective parameters and characteristics of dielectric antennas	[29]
2017	An application-oriented detailed survey of DR antennas in the last three and half decades is addressed.	[30]
2017	A survey of various CP radiation methods for DR antennas is comprehensively discussed.	[31]
2019	A review of bandwidth improvement methods is highlighted and discussed in detail.	[32]
2020	Design advances and trends in various types of CP antennas are studied comprehensively	[33]

**Table 2:** Performance analysis of wideband DR-based CP hybrid antennas.

HDRA Description [Reference]	CP is Achieved by	Volume of the HDRA (in Terms of λ at f_r_) (L × W × H_eff_)	f_r_ (or) CP Bands of Resonance (GHz)	10 dB RL Bandwidth (MHz) or %	3 dB AR Bandwidth (MHz) or %	Gain (dBic)
Four dielectric inserts under the patch and coupled with cross slot [109]	Dielectric inserts and square patch coupled through cross-slot	0.67λ × 0.67λ × 0.075λ	L-band	7.8 %	2.5%	9.1
A strip-line fed rectangular DRA with top-loaded rectangular patch [110]	Rectangular DR element loaded with a rectangular patch with various aspect ratios	0.37λ × 0.37λ × 0.048λ	2170–2270	100	25 or 1.1%	3.3
Cylindrical DRA and ground plane having four slots fed through microstrip line feed network [111]	Having a feeding network consisting of four microstrip lines; wherein the four slots are constructed and geometrically arranged to ensure CP	0.8λ × 0.8λ × 0.12λ	1.08–1.82	740	600	5
Four sequentially rotated arc-shaped slots etched in the ground plane to feed the DRA [112]	Due to the arc-shaped slots	0.8λ × 0.8λ × 0.118λ	1.22- 1.71	490	380	3
Aperture coupled microstrip loaded with an elliptical ring dielectric resonator [113]	A reversed T-shaped coupling slot	6λ × 4λ × 0.252λ	55.6–65	9400	2000	9
Rectangular DRA with modified slot and microstrip line [114]	Modified cross-slot	0.43λ × 0.43λ × 0.29λ	2.19–2.92	730	630	5
An open-ended slot with Stair-shaped DR loaded on the ground [115]	Combination of Stair-shaped DR and Open-ended slot on the ground plane with an offset feed	0.46λ × 0.46λ × 0.07λ	3.844–8.146	4302	2480	3.9
Cylindrical DR loaded on L shaped microstrip line with vertical strips-lines attached to DR [116]	Dual vertical microstrip lines with L-shaped microstrip-line arranged perpendicularly to excite orthogonal modes	0.59λ × 0.59λ × 0.26λ	2.82–3.83	1010	770	5.5
A rectangular DRA with conformal metal strip [117]	Employment of parasitic patch at an optimized distance beside the conformal metal strip of the two identical rectangular DRAs to generate CP	0.46λ × 0.46λ × 0.34λ	3.50–4.95	1450	820	6.2

**Table 3:** Performance analysis of multi-functional DR-based CP hybrid antennas.

HDRA Description [Reference]	CP is Achieved by	Volume of the HDRA (in Terms of λ at Lower f_r_) (L × W × H_eff_)	f_r_ or CP Bands of Resonance (GHz)	10 dB RL Bandwidth (MHz)	3 dB AR Bandwidth (MHz)	Gain (dBi)
A zonal-slot antenna cut onto a conducting cavity is combined with rectangular DRA [120]	Zonal and cross slots with L-Probe feed ensures CP	0.275λ × 0.275λ × 0.26λ	2.34–2.53, and 4.46–5.34	190, and 880	80, and 180	5.80 and 4.29
A cross-slot acts as both the feeding structure of the DRA and an effective radiator [121]	Cross slot as aperture coupled feed and radiator	0.475λ × 0.475λ × 0.098λ	1.80–2.07, and 2.57–2.92	270, and 350	60, and 100	4.7, and 5.6
Consists of a Z-shaped CDRA along with a dual C-shaped patch [122]	Offset between upper and lower CDRAs is responsible for CP	0.22λ × 0.22λ × 0.049λ	7.2–8.5	1300	500	2.5, 3.2, 3.5, 4 and 6

**Table 4:** Performance analysis of dual-sense polarized DR-based hybrid antennas.

HDRA Description [Reference]	Volume of the HDRA (in Terms of λ at Lower f_r_) (L × W × H_eff_)	Dual-Sense Polarized Bands	f_r_ (GHz) or CP Band of Resonance	10 dB RL Bandwidth (MHz)	3 dB AR Bandwidth (MHz)	Gain (dBi)
A ring-shaped patch along with an inverted L-strip and cylindrical DRA [126]	0.475λ × 0.475λ × 0.098λ	LP and CP	2.9–3.93	1030	250	4
Comprises of an asymmetrical square ring-shaped printed line and a rectangular DR. The square ring is responsible for creating dual-sense radiation [127]	0.68λ × 0.57λ × 0.11λ	LHCP and RHCP	3.28–5.78	2500	470 and 300	3.1
Modified hexagonal DR is top-loaded with a square microstrip ring [128]	0.44λ × 0.51λ × 0.152λ	LHCP and RHCP	1.75–2.03, 2.23–2.96, and 3.65–3.76	280, 730 and 110	70, 150 and 80	5, 5.28 and 2.36
The asymmetric-slot radiator is fed by an L-shaped stub with the CPW line combined with rectangular-DR. Dual sense CP is obtained using a rectangular-DR over asymmetric rectangular-slot radiator with an L-shaped feed line [129]	0.446λ × 0.446λ × 0.149λ	LHCP and RHCP	1.75–2.73	980	860	5.5

## Data Availability

Not applicable.
